# Analysis of Anxiety and Depression Status in Patients Undergoing Radiotherapy During the COVID-19 Epidemic

**DOI:** 10.3389/fpsyt.2021.771621

**Published:** 2021-12-10

**Authors:** Liping Yang, Jing Yang, Jian He, Yan Zhou, Yangyang Zhang, Bin Sun, Jing Gao, Liting Qian

**Affiliations:** Department of Radiotherapy Oncology, The First Affiliated Hospital of USTC, Division of Life Sciences and Medicine, University of Science and Technology of China, Hefei, China

**Keywords:** COVID-19, cancer patients, anxiety, depression, stressors

## Abstract

**Background:** The 2019 coronavirus (COVID-19) had caused a global pandemic and disrupted millions of lives. Cancer patients are a special group at greater risk of contracting viruses. This study aimed to evaluate the anxiety and depression status of cancer patients undergoing radiotherapy during the COVID-19 epidemic.

**Methods:** 396 cancer patients who underwent radiotherapy were enrolled in this study. The self-rating anxiety scale (SAS) and self-rating depression scale (SDS) were used to evaluate patient anxiety and depression, respectively. 373 cancer patients completed the questionnaires.

**Results:** During the COVID-19 outbreak, the incidence of anxiety and depression in cancer patients were 34.9 and 33.8%, respectively. Approximately 31.4% of tumor radiotherapy patients had anxiety and depression. Based on univariate analysis, age, work status, education level, and clinical stage were related to anxiety and depression in cancer patients. Based on multiple regression analysis, age and clinical stage were related to anxiety, but only age was related to depression.

**Conclusions:** Due to the COVID-19 pandemic, cancer patients experienced increased psychological problems. Our results have contributed to a better understanding of these psychological problems in cancer patients and provide a basis for psychological counseling and intervention.

## Introduction

Since COVID-19 was first reported in Wuhan, Hubei Province, China (1), COVID-19 had spread rapidly worldwide. COVID-19 has high pathogenicity, virus specificity, and mobility. COVID-19 had been upgraded from a regional public health emergency to a global public crisis (2) that threatened the safety of human life and social stability. In January 2020, the World Health Organization (WHO) defined the virus that caused severe pneumonia as the 2019 novel coronavirus (COVID-19) (3). In March 2020, the WHO announced that COVID-19 had caused a global pandemic (4). In October 2020, Delta, a new lineage of the COVID-19 virus was discovered in the Indian. In July 2021, WHO declared the Delta variants were becoming the major popular strain in many countries. As of August 2021, the cumulative number of new coronavirus infections had exceeded 219 million worldwide, and the cumulative number of deaths were approximately 4.55 million (https://covid19.who.int/). Affected by the deterioration of the epidemic, many countries had adopted measures, such as quarantines, suspension of work, curfews, cancellation of large gatherings, blockades, and other measures, to curb the further spread of the epidemic. The outbreak of the disease and strict control measures had landed a heavy blow to the medical system and economy (5). Specifically, our work, lives, social roles and functions had been affected that in turn caused a series of psychological problems related to COVID-19, including anxiety, depression, stress, and insomnia, which had caught the attention in caused social and medical circles. Shi et al. (6) collected 2651 chinese people, they investigated the anxiety and depression levels of the general population during the COVID-19 pandemic in China. They found that lower knowledge poorer health perceiving higher risks of infections unstable working condition presented higher anxiety and depression levels, while female married lived alone were more anxious; living areas was not related to anxiety and depression. Shehata et al. (7) reported that the depression was correlated with age mental status and being infected with COVID-19, while only age correlated with anxiety.

Cancer patients are a special group. Studies (8) have shown that the anxiety and depression rates are higher than in healthy people. Of cancer patients, 17–46% have anxiety and depression (9). As a result of the tumor and treatment, many patients with long-term malnutrition and low immunity are more susceptible to virus infection (10, 11). Given the current pandemic, the normal treatment of cancer patients might be interrupted or changed, and the social support was limited (12, 13). Thus, cancer patients are more likely to be troubled by psychological problems. Currently, little attention is paid to the psychological problems of cancer patients caused by COVID-19, thus it is particularly important to pay attention to and ease the anxiety and depression of cancer patients in a timely manner.

The purpose of this study was to evaluate the anxiety and depression status of cancer patients undergoing radiotherapy during the COVID-19 epidemic, and to analyze the factors that influence anxiety to depression to provide a basis for psychological counseling and intervention.

## Materials and Methods

### Participants

396 patients who underwent radiotherapy in the Department of Radiotherapy at Anhui Cancer Hospital from August 2020 to August 2021 were enrolled in this cross-sectional study. The inclusion criteria were as follows: (1) age ≥18 years; (2) diagnosed with a tumor and scheduled to receive radiotherapy in the upcoming days/weeks; (3) able to read and communicate with informed consent. The exclusion criteria were as follows: (1) Previous severe mental illness or a combination of disorders that may affect anxiety or depression, such as schizophrenia, autism, delusion, obsessive-compulsive disorder, hypothyroidism, etc.; (2) cognitive and communication disorders; (3) Recently suffered a major trauma; (4) and declined to join the group.

This study was approved by the Human Ethics Review Committee of Anhui Provincial Cancer Hospital Center, China. All patients signed an informed consent before the study commenced.

### Investigation Tools and Approach

This study was based on a questionnaire survey, for which the participants provided informed consent. We collected clinical data, including age, gender, education level, occupation, marital status, place of residence, cigarette smoking and alcohol consumption histories, clinical staging, and distant metastases. We adopted a self-rating anxiety scale (SAS) and self-rating depression scale (SDS) to evaluate anxiety and depression, respectively.

The SAS was developed by Zung (14) as a psychological scale for assessing the degree and changes in anxiety. A 4-level scoring system was used to assess patients. The 4-level scoring included 20 items, 5 of which had reverse scoring. The main assessment item was the frequency of symptoms such as fear, panic, madness, tremor of hands and feet, body pain, palpitations, dizziness, dyspnea, frequent urination, hyperhidrosis and sleep disturbance, etc. The scoring method was based on 1–4 points, as follows: (1) no or a very limited amount of time; (2) a small amount of time; (3) a considerable amount of time; and (4) most or all of the time. The scores of the 20 items were added and multiplied by 1.25 to obtain the integer of the standard points. The score was divided by 50; 50–59 was mild anxiety, 60–69 was moderate anxiety, and >70 was severe anxiety. The SDS, which is intuitive and concise, was created by Zung (15) in 1965. The SDS is a psychological scale for assessing the degree and change in depression, Including two items of mental-emotional symptoms, eight items of physical disorder, two items of psychomotor disorder, and eight items of depressive psychological disorder. The SDS also uses 4 levels of scoring with a total of 20 items, of which 10 items are reverse scored. The calculation of the standard score is similar to the SAS score. The scores of 20 items are added, then multiplied by 1.25 to determine the integer. The SDS score cut-off value is 53; 53–62 is mild depression, 63–72 is moderate depression, and > than 73 is severe depression.

In the current study the Cronbach coefficients of the SAS and SDS scales were 0.856 and 0.874, respectively. A total of 396 questionnaires were distributed, of which 373 valid questionnaires were returned.

### Statistical Analysis

The statistical analyses were performed using version 23.0 of the Statistical Product and Service Solutions (SPSS; International Business Machines, Corp., Armonk, NY, USA). Continuous variables are represented by the means ± SD. Normally distributed data were tested by the Shapiro-Wilk test. Independent *t*-tests were used for comparison between the two groups, single-factor analysis of variance was used for comparison between multiple groups, and multivariate analysis was performed by multiple regression analysis. A two-tailed *P* <0.05 was considered statistically significant.

## Results

### Patient Characteristics

From August 2020 to August 2021 there were 396 patients undergoing radiotherapy in our department, of whom 10 interrupted radiotherapy and 13 could not communicate effectively to complete the questionnaire. A total of 373 patients undergoing radiotherapy were enrolled in this study, including 245 males and 128 females. The median age was 57 years (range, 22–89 years). The tumor types were as follows: 81 cases of esophageal cancer; 57 cases of nasopharyngeal cancer; 13 cases of oropharyngeal cancer; 17 cases of hypopharynx cancer; 13 cases of oral cancer; 49 cases of intracranial tumors; 18 cases of lung cancer; 27 cases of breast cancer; 33 cases of laryngeal cancer; 27 cases of rectal cancer; 9 cases of gastric cancer; 6 cases of thyroid cancer; 13 cases of nasal cavity NK/T cell lymphoma; 3 case of endometrial cancer; and 7 cases of other tumors types.

### Anxiety and Depression in Patients Undergoing Radiotherapy

The mean anxiety score of tumor radiotherapy patients was 45 (range, 25–73) and the mean depression score was 49 (range, 25–77). The incidence of anxiety and depression was 34.9 and 33.8%, respectively. Among the tumor radiotherapy patients, 31.4% had both anxiety and depression, as shown in Table 1.

**Table 1:** The results of anxiety and depression in 373 tumor radiotherapy patients during COVID-19 pandemic.

**Project**	**Anxiety cases (%)**	**Depression cases (%)**
**Degree**		
None	243 (65.1%)	247 (66.2%)
Mild	77 (20.6%)	63 (16.9%)
Moderate	52 (13.9%)	49 (13.1%)
Severe	1 (0.4%)	14 (3.8%)
Both anxiety and depression	117 (31.4%)	117 (31.4%)

### Univariate Analysis of Anxiety and Depression in Tumor Radiotherapy Patients

Age, gender, education level, occupation, marital status, residence, cigarette smoking and alcohol consumption histories, clinical stage, and distant metastases were included in the univariate analysis. The results suggested that anxiety and depression were related to age, work status, education level, and clinical stage (all, *P* < 0.05), as shown in Table 2.

**Table 2:** Univariate analysis of anxiety and depression in 373 tumor radiotherapy patients.

**Characteristic**	**cases**	**Anxiety**	***P* value**	**Depression**	***P* value**
		**score**		**score**	
**Gender**					
Male	245	45.84 ± 10.23	0.426	50.02 ± 11.11	0.686
Female	128	44.63 ± 10.81		48.15 ± 11.64	
**Age**					
≤ 40years	36	35.19 ± 8.55	0.0006	42.08 ± 9.39	0.0001
40–60years	173	41.56 ± 8.51		45.04 ± 9.00	
≥60years	164	51.07 ± 9.83		55.55 ± 10.84	
**Marriage**					
Married	348	44.47 ± 9.62	0.102	48.30 ± 10.40	0.117
Divorced/Single/ Widowed	25	58.76 ± 12.22		64.32 ± 13.07	
**Working status**					
Stable work	129	40.21 ± 9.43	0.0001	43.78 ± 10.12	0.0001
Retirement	59	48.03 ± 9.82		52.17 ± 10.50	
No work/Temporary Worker	185	48.23 ± 9.93		52.39 ± 10.91	
**Education level**					
Primary school	137	50.15 ± 10.87	0.0001	54.39 ± 11.87	0.0001
High school/Middle school	197	43.93 ± 8.89		47.76 ± 9.60	
University	39	36.36 ± 7.66		39.92 ± 8.76	
**Place of residence**					
City	191	42.02 ± 10.34	0.377	46.73 ± 11.08	0.185
Rural area	182	47.95 ± 9.94		52.16 ± 10.91	
**Smoking**					
Yes	69	46.75 ± 11.18	0.210	51.51 ± 11.81	0.234
No	304	45.12 ± 10.25		48.89 ± 11.16	
**Drinking**					
Yes	61	46.54 ± 10.95	0.365	51.23 ± 11.54	0.407
No	312	45.16 ± 10.32		48.98 ± 11.25	
**Clinical stage**					
I	14	33.43 ± 4.67	0.0001	37.07 ± 5.41	0.0001
II	82	42.01 ± 9.59		45.45 ± 10.61	
III	158	45.73 ± 9.88		49.64 ± 10.51	
IV	119	48.87 ± 10.50		53.29 ± 11.45	
**Distant metastasis**					
Yes	31	54.06 ± 10.33	0.627	59.03 ± 11.14	0.508
No	342	44.64 ± 10.10		48.50 ± 10.93	

### Multiple Regression Analysis of Anxiety and Depression in Tumor Radiotherapy Patients

We entered age, work status, education level, and clinical stage, which were statistically significant factors in univariate analysis, into multiple regression analysis as independent variables; anxiety and depression scores were dependent variables. Age and clinical stage were related to anxiety, and age was related to depression (all, *P* < 0.05), as shown in Table 3.

**Table 3:** Multiple regression analysis of anxiety and depression in tumor radiotherapy patients.

**Independent**	**Unstandardized**	**Standard**	**Standardization**	***P* value**
**variable**	**coefficient**	**error**	**factor**	
**Anxiety**				
Age	4.701	0.804	0.292	0.0001
Stage	3.356	0.540	0.268	0.0001
**Depression**				
Age	5.064	0.874	0.290	0.0001

## Discussion

The COVID-19 pandemic is a high-stress event, which has had a tremendous impact and pressure on economic development, social stability, and the physical and mental health of people in all countries (16–19). Studies (9, 16, 19) have shown that in the context of the current pandemic, cancer patients had a significantly higher degree of anxiety and depression than healthy people and were more likely to be troubled by mental illness. The factors influencing anxiety and depression in tumor radiotherapy patients are complex and unclear. The current study determined the prevalence and factors influencing anxiety and depression in cancer patients receiving radiotherapy during the novel coronavirus epidemic.

In this study, the patients anxiety and depression were evaluated by using the Chinese version of SDS and SAS with the Cronbach coefficient of 0.784 for SDS in Chinese people (20), and of 0.931 for SAS in the general population (21). The results showed that they had good reliability, validity and excellent internal consistency. Wang et al. (22) studied on the public psychological states and its related factors during the COVID-19 outbreak by using the Chinese version of SDS and SAS; Song et al. (23) investigated the mental health status of college students under the COVID-19 pandemic and explored potential influential factors, they used the Chinese version of SDS and SAS to evaluate the anxiety and depression of college students.

Ma et al.^.^(24) reported that the age of tumor radiotherapy patients >60 years of age were more prone to anxiety, and age was not a factor that affected depression. The results were similar to the results of our study. In this study age ≥60 years was a factor influencing anxiety and depression in tumor radiotherapy patients. Elderly cancer patients often have chronic underlying diseases for a long time and are prone to anxiety and depression (25). Many elderly cancer patients live apart from their children and have limited medical knowledge and information, due to a lack of understanding regarding the new coronavirus disease, the elderly are prone to anxiety, panic attacks, or helpless despair (26). In addition, due to the impact of the epidemic, many hospitals have adopted online appointments, diagnosis, and treatment. Elderly tumor patients are often not proficient in operating such a network system, which is inconvenient when seeking medical treatment, thus leaving the patient feeling helplessness or isolation, which in turn might cause the patient to experience anxiety and depression or even aggravate existing anxiety and depression (24).

In our study, the results of Univariate analysis showed that work status and the education level of tumor radiotherapy patients were related to anxiety and depression. We divided the work status of the enrolled patients into stable work, retired, and no or temporary work, and found that the incidence of anxiety and depression in patients with stable work or income was significantly lower than patients without work or no stable income (both *p* = 0.0001). We speculate that the reason for this finding might be that cancer patients need to spend a considerable amount of money to receive treatment, which requires family financial support, and these expenditures create a large economic burden on families without a job or a stable source of income. In addition, because of the epidemic, the social economy has been greatly affected. Many small companies have broken their capital chains or even gone bankrupt. The number of unemployed people has increased sharply, and economic income has declined, which has also increased anxiety and depression in cancer patients. Shi et al. (27) reported that anxiety and depression are not related to education level. Ma et al. (24) also reported similar findings due to the small proportion of enrolled patients with a higher education. In our study, 197 patients had a middle school education and above and 39 patients had a University education. The incidence of anxiety and depression were negatively correlated with the level of education in tumor radiotherapy patients. The lower the level of education, the more likely a patient was to be anxious and depressed. We speculate that the possible reason for this association is that patients with a higher education level have more ways to receive information and have a strong ability to discriminate and process information. Patients with a higher education level have a better understanding of cancer, especially in the context of the COVID-19 epidemic, which has been highly infectious and fatal. Patients with higher education levels could better understand COVID-related knowledge and did not panic blindly, thereby avoiding or reducing anxiety and depression. But in Multiple regression analysis, work status and the education level of tumor radiotherapy patients were not related to anxiety and depression. It is necessary to expand the sample size for further research.

In addition, our study showed that patients with an advanced clinical stage had a higher incidence of anxiety and depression. As a reliable indicator of prognosis, clinical staging is related to the histologic type and grade of tumors, which can predict the biological behavior of malignant tumors and provide a basis for clinicians to accurately select treatment options (28).

Advanced clinical staging is often related to more severe clinical symptoms or tumor invasion, which are associated with a poor prognosis. Tumor patients with advanced clinical stages often have anxiety and depression because they are worried about tumor progression, metastasis, and an unsatisfactory treatment effect (29).

Our research had some limitations. First, the number of patients we enrolled was limited and the sample size was not big, which had an impact on the results of the study. We calculated the sample size by using the G^*^Power calculator (if α = 0.05, 1-β = 0.9, *n* = 404). Second, we collected cancer patients who underwent radiotherapy in this study. The toxic side effects of the patients due to radiotherapy might aggravate depression and anxiety, while depression and anxiety of these patients might at their peak during radiotherapy which had an impact on the results. Third, our study included cancer patients with all tumor types, and the results were overall results which couldn't reflect the anxiety and depression of patients with different types of tumors. Fourth, the gender distribution of our study was biased toward males, this also affect the results. Fifth, this was a cross-sectional study, which limited the inferences of the study. It is especially necessary to expand the sample size, perform subgroup analysis for different types of tumors, continue investigations at each node of the epidemic, and combine longitudinal analysis of the anxiety and depression status of tumor radiotherapy patients.

## Conclusion

In conclusion, the present study suggested that during the COVID-19 epidemic, the age and clinical stage were related to anxiety with patients undergoing radiotherapy, but only age was related to depression. We hope our results may contributed to cancer patients for psychological counseling and intervention.

## Data Availability Statement

The original contributions presented in the study are included in the article/supplementary material, further inquiries can be directed to the corresponding author/s.

## Ethics Statement

The studies involving human participants were reviewed and approved by the Human Ethics Review Committee of Anhui Provincial Cancer Hospital Center, China. The patients/participants provided their written informed consent to participate in this study.

## Author Contributions

LQ, JG, LY, and JY conceived and designed the study. JY, JH, YZho, YZha, and BS performed the study. LY analyzed the data. LY, JY, LQ, and JG wrote the manuscript. LQ, JG, and LY constructive discussions. All authors contributed to the article and approved the submitted version.

## Conflict of Interest

The authors declare that the research was conducted in the absence of any commercial or financial relationships that could be construed as a potential conflict of interest.

## Publisher's Note

All claims expressed in this article are solely those of the authors and do not necessarily represent those of their affiliated organizations, or those of the publisher, the editors and the reviewers. Any product that may be evaluated in this article, or claim that may be made by its manufacturer, is not guaranteed or endorsed by the publisher.

## References

[1] Zhu N Zhang D Wang WL Li XW Yang B Song JD. A novel coronavirus from patients with pneumonia in China, 2019. N Engl J Med. (2020) 382:727–33. 10.1056/NEJMoa2001017 31978945 PMC7092803

[2] TM Barber. COVID-19 and diabetes mellitus :implications for prognosis and clinical management. Expert Rev Endocrinol Metab. (2020) 15:227–36. 10.1080/17446651.2020.1774360 32511033

[3] Vetter P Vu DL L'Huillier AG Schibler M Kaiser L Jacquerioz F. Clinical features of covid-19. BMJ. (2020) 369:m1470. 10.1136/bmj.m1470 32303495

[4] Cucinotta D Vanelli M. Who declares COVID-19 a pandemic. Acta Biomed. (2020) 91:157–60. 10.23750/abm.v91i1.9397 32191675 PMC7569573

[5] Lai CC Wang CY Wang YH Hsueh SC Ko WC Hsueh PR. Global epidemiology of coronavirus disease 2019 (COVID-19): disease incidence, daily cumulative index, mortality, and their association with country healthcare resources and economic status. Int J Antimicrob Agents. (2020) 55:105946. 10.1016/j.ijantimicag.2020.105946 32199877 PMC7156123

[6] Shi ZY Qin YL Chair SY Liu YH Tian Y Li X. Anxiety and depression levels of the general population during the rapid progressing stage in the coronavirus disease 2019 outbreak: a cross-sectional online investigation in China. BMJ Open. (2021) 11:e050084. 10.1136/bmjopen-2021-050084 34011604 PMC8137187

[7] Shahata GA Gabra R Eleellawy S Elsayed M Gaber DE Elshabrawy HA. Assessment of anxiety, depression, attitude, and coping strategies of the Egyptian population during the COVID-19 Pandemic. J Clin Med. (2021) 10:3989–98. 10.3390/jcm10173989 34501437 PMC8432513

[8] Yang YL Liu L Wang Y Wu H Yang XH Wang JN. The prevalence of depression and anxiety among Chinese adults with cancer a systematic review and meta-analysis. BMC Cancer. 2013:13:393. 10.1186/1471-2407-13-393 23967823 PMC3765872

[9] Nikbakhsh N Moudi S Abbasian S Khafri S. Prevalence of depression and anxiety among cancer patients. Caspian J Intern Med. (2014) 5:167–70. 25202445 PMC4143739

[10] Li JJ Santa-Maria CA Feng HF Wang LC Zhang PC Xu YB. Patient-reported outcomes of patients with breast cancer during the COVID-19 outbreak in the epicenter of China: a cross-sectional survey study. Clin Breast Cancer. (2020) 20:e651–2. 10.1016/j.clbc.2020.06.003 32709505 PMC7275993

[11] Lai SJ Ruktanonchai NW Zhou LC Proper O Luo W Floyd JR. Effect of non-pharmaceutical interventions to contain COVID-19 in China. Nature. (2020) 585:410–3. 10.1038/s41586-020-2293-x 32365354 PMC7116778

[12] Burki TK. Cancer guidelines during the COVID-19 pandemic. Lancet Oncol. (2020) 21:629–30. 10.1016/S1470-2045(20)30217-5 32247319 PMC7270910

[13] Tagliamento M Spagnolo F Poggio F Soldato D Conte B Ruelle T. Italian survey on managing immune checkpoint inhibitors in oncology during COVID-19 outbreak. Eur J Clin Invest. (2020) 50:e13315. 10.1111/eci.13315 32535890 PMC7323025

[14] Zung WW. A rating instrument for anxiety disorders. Psychosomatics. (1971) 12:371–9. 10.1016/S0033-3182(71)71479-0 5172928

[15] Zung WW. A self-rating depression scale. Arch Gen Psychiatry. (1965) 12:63–70. 10.1001/archpsyc.1965.01720310065008 14221692

[16] Romito F Dellino M Loseto G Opinto G Silvestris E Coormio C. Psychological distress in outpatients with lymphoma during the COVID-19 pandemic. Front Oncol. (2020) 10:1–6. 10.3389/fonc.2020.01270 32754447 PMC7365920

[17] Wang CY Pan R Wan XY Tan YL Xu LK Ho CS. Immediate psychological responses and associated factors during the initial stage of the 2019 coronavirus disease (COVID-19) epidemic among the general population in China. Int J Environ Res Public Health. (2020) 17:1729. 10.3390/ijerph17051729 32155789 PMC7084952

[18] Petzold MB Plag J Ströhle A. COVID-19-Pandemie Psychische Belastungen können reduziert werden. Deutsches Ärzteblatt. (2020) 117:648–54. Available online at: https://www.aerzteblatt.de/archiv/213283/COVID-19-Pandemie-Psychische-Belastungen-koennen-reduziert-werden

[19] Cullen W GulatiG Kelly BD. Mental health in the COVID-19 pandemic. QJM. (2020) 113:311–2. 10.1093/qjmed/hcaa110 32227218 PMC7184387

[20] Peng H Zhang YY Ji Y Tang WQ Li Q Yan XL. Analysis of reliability and validity of Chinese version SDS scale in women of rural area. Shanghai Med 18Pharmaceutical J. (2013) 34:20–3. 10.3969/j.issn.1006-1533.2013.14.011

[21] Hühne A Welsh DK Landgraf D. Prospects for circadian treatment of mood disorders. Ann Med. (2018) 50:637–54. 10.1080/07853890.2018.1530449 30265156

[22] Wang YN Di Y Ye YJ Wei WB. Study on the public psychological states and its related factors during the outbreak of coronavirus disease 2019 (COVID-19) in some regions of China. Psychol Health Med. (2021) 26:13–22. 10.1080/13548506.2020.1746817 32223317

[23] Song HT Ge CH Chang LX Zhao TT WU W Ge DX. Investigation on the psychological status of college students during the coronavirus disease-2019 epidemic. J Gen Psychol. (2021) 12:11–2. 10.1080/00221309.2021.1893637 33709883

[24] Ma JB Zhen HN Guan H Liu ZK Jing S Wang WH. Analysis of anxiety and depression in patients undergoing radiotherapy during COVID-19 epidemic period. Chin J Radiat Oncol. (2020) 29:615–8. 10.3760/cma.j.cn113030-20200417-00188

[25] Wen SS Xiao HM Yang YQ. The risk factors for depression in cancer patients undergoing chemotherapy a systematic review. Support Care Cancer. (2019) 27:57–67. 10.1007/s00520-018-4466-9 30225571

[26] Qin T Hu T Ge QQ Zhou XN Li JM Jiang CL. COVID-19 pandemic related long-term chronic stress on the prevalence of depression and anxiety in the general population. BMC Psychiatry. (2021) 21:380–90. 10.1186/s12888-021-03385-x 34320924 PMC8316891

[27] Shi P Liu Z Zhang YB J Wang BN Hui Wang T. Novel coronavirus pneumonia current situation and influencing factors of anxiety in cancer patients. J Mod Oncol. (2020) 28:1608–10. 10.3969/j.issn.1672-4992.2020.09.044

[28] Rosen RD Sapra A. TNM Classification. Statparls. (2021) 1:e31985980.

[29] Massicotte V Ivers H Savard J. COVID-19 Pandemic stressors and psychological symptoms in breast cancer patients. Curr Oncol. (2021) 28:294–300. 10.3390/curroncol28010034 33430131 PMC7903274

