# Corrigendum: Analysis of Anxiety and Depression Status in Patients Undergoing Radiotherapy During the COVID-19 Epidemic

**DOI:** 10.3389/fpsyt.2022.912496

**Published:** 2022-04-26

**Authors:** Liping Yang, Jing Yang, Jian He, Yan Zhou, Yangyang Zhang, Bin Sun, Jing Gao, Liting Qian

**Affiliations:** Department of Radiotherapy Oncology, The First Affiliated Hospital of USTC, Division of Life Sciences and Medicine, University of Science and Technology of China, Hefei, China

**Keywords:** COVID-19, cancer patients, anxiety, depression, stressors

In the published article, there was an error in the affiliation. Instead of “Division of Life Sciences and Medicine, Department of Radiotherapy Oncology, The First Affiliated Hospital of USTC, University of Science and Technology of China, Hefei, China,” it should be “Department of Radiotherapy Oncology, The First Affiliated Hospital of USTC, Division of Life Sciences and Medicine, University of Science and Technology of China, Hefei, China.”

The authors apologize for this error and state that this does not change the scientific conclusions of the article in any way. The original article has been updated.

## Publisher's Note

All claims expressed in this article are solely those of the authors and do not necessarily represent those of their affiliated organizations, or those of the publisher, the editors and the reviewers. Any product that may be evaluated in this article, or claim that may be made by its manufacturer, is not guaranteed or endorsed by the publisher.

